# Transcriptome Analyses in Different Cucumber Cultivars Provide Novel Insights into Drought Stress Responses

**DOI:** 10.3390/ijms19072067

**Published:** 2018-07-16

**Authors:** Min Wang, Biao Jiang, Qingwu Peng, Wenrui Liu, Xiaoming He, Zhaojun Liang, Yu’e Lin

**Affiliations:** 1 Vegetable Research Institute, Guangdong Academy of Agricultural Sciences, Guangzhou 510640, China; w.jsun@163.com (M.W.); jiangbiao198354@163.com (B.J.); pengqingwu@gdaas.cn (Q.P.); liuwr10@126.com (W.L.); xiaominghe626@163.com (X.H.); liangzhaojun@gdaas.cn (Z.L.); 2 Guangdong Key Laboratory for New Technology Research of Vegetables, Guangzhou 510640, China

**Keywords:** *Cucumis sativus* L., RNA-Seq, DEGs, sucrose, ABA, drought stress

## Abstract

Drought stress is one of the most serious threats to cucumber quality and yield. To gain a good understanding of the molecular mechanism upon water deficiency, we compared and analyzed the RNA sequencing-based transcriptomic responses of two contrasting cucumber genotypes, L-9 (drought-tolerant) and A-16 (drought-sensitive). In our present study, combining the analysis of phenotype, twelve samples of cucumber were carried out a transcriptomic profile by RNA-Seq under normal and water-deficiency conditions, respectively. A total of 1008 transcripts were differentially expressed under normal conditions (466 up-regulated and 542 down-regulated) and 2265 transcripts under drought stress (979 up-regulated and 1286 down-regulated). The significant positive correlation between RNA sequencing data and a qRT-PCR analysis supported the results found. Differentially expressed genes (DEGs) involved in metabolic pathway and biosynthesis of secondary metabolism were significantly changed after drought stress. Several genes, which were related to sucrose biosynthesis (*Csa3G784370* and *Csa3G149890*) and abscisic acid (ABA) signal transduction (*Csa4M361820* and *Csa6M382950*), were specifically induced after 4 days of drought stress. DEGs between the two contrasting cultivars identified in our study provide a novel insight into isolating helpful candidate genes for drought tolerance in cucumber.

## 1. Introduction

Drought stress generally occurs when soil water is deficient, leading to a continuous loss of water by transpiration or evaporation [1]. Water deficiency, a key limiting factor in plant growth and development, impacts plant elongation and expansion growth [2,3]. In order to survive under drought stress, plants have to make corresponding adjustments by regulating gene expression of stress-related and signal transduction pathways [4,5,6], such as reactive oxygen species (ROS)-related genes [7], transcription factors (TFs) [8], and the abscisic acid (ABA) signal transduction pathway [9,10].

Cucumber (*Cucumis sativus* L.), one of the most important vegetable crops in Cucurbitaceae, is originally from the southern Himalayas and shows a preference for warm and moist environment [11]. Previous studies about cucumber resistance on drought have been carried out in different aspects [12,13,14,15]. Carbon monoxide (CO) is involved in hydrogen gas (H_2_)-induced adventitious root development under stimulated drought stress and alleviates oxidative damage by altering relative physiological index [12]. *CsCER1* is involved in the fruit cuticle synthesis, and overexpressing the gene has been shown to improve the drought tolerance under water-deficiency conditions [13]. Exogenously applied hydrogen peroxide could considerably enhance the cucumber drought resistance by increasing the plant’s antioxidative defense system and its capacity for osmotic adjustment [14]. Tobacco PR-2d promoter/*uidA* (GUS) gene is induced in transgenic cucumber and improves the response to biotic and abiotic stimuli [15].

Comparing transcriptome by RNA-seq of various genotypes in different species is one of the most suitable techniques for exploring resistant genes under abiotic stress and elucidating the role of various biological pathways, as well as mechanisms for influencing tolerance to adverse environments [16,17]. When compared with microarray and expressed sequence tag, advantages of RNA-seq showed determination of alternative splicing (AS) events, novel transcripts and digital gene expression at the isoform level [18,19]. In cucumber, the RNA-seq method has been widely employed for performing crucial agricultural functions such as fruit development [20], parthenocarpy [21], flower sex expression [22], and other plant responses to abiotic stresses [23,24,25]. A transcriptome profiling reveals a mechanism of fruit trichome formation, which plays an important role in plant defense against biotic and abiotic stresses [23]. A total of 121 genes were significantly induced under melatonin treatment, which promoted the cucumber lateral root formation under salt stress [24]. Zhao et al. [25] examined over 23,000 transcripts in cucumber leaves, and found that 364 genes were differentially expressed in response to nitrogen deficiency, providing novel insights into the responses of cucumber to N starvation at the global transcriptome level [25]. However, to the best of our knowledge, no research has been performed on the drought stress in cucumber using compared transcriptome.

In this study, we carried out RNA-sequencing analysis in cucumber to explore the transcriptional variations between a drought-tolerant cultivar L-9 and a drought-sensitive cultivar A-16 under normal and drought conditions. Different drought stress-responsive novel transcript isoforms were identified between L-9 and A-16. Furthermore, we analyzed the differential gene expression patterns in response to drought stresses. Functional categorization of differentially expressed transcripts was carried out to reveal various metabolic pathways involved in drought responses. Overall, this study provides a theoretical basis for further study of the regulatory mechanism of drought tolerance in cucumber.

## 2. Results

### 2.1. A-16 Cultivar Is Sensitive to Drought Stress

**Figure 1:**
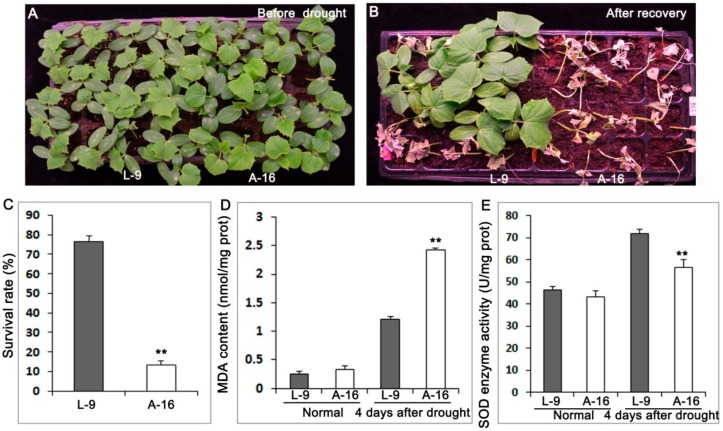
Phenotypes of L-9 and A-16 before drought and after recovery of drought stress. (**A**) L-9 and A-16 plants were grown under normal conditions for 14 days. (**B**) After 7 days drought treatment, seedlings recovered for 3 days. (**C**) Survival rate of plants following the 7-day drought treatment. (**D**,**E**) Measurement of MDA content (**D**) and SOD enzyme activity (**E**) under normal conditions and 4 days after drought. Data is presented as the mean ± standard deviation (*n* = 9). ** *p* < 0.01; Student’s *t*-test.

Ten-day seedlings of L-9 and A-16 (120 plants for three biological replicates, respectively) grown under normal condition (Figure 1A) were treated with water deficiency for 7 days and recovered for 3 days (Figure 1B). Both L-9 and A-16 showed vigorous development before drought; however, A-16 began to exhibit wilting at the top of the growth point after drought stress, and its leaves turned chlorotic and yellow (Figure 1A,B). Approximately 13% of the drought treated A-16 plants survived after the subsequent 3-day recovery, compared with 77% of L-9 plants (Figure 1C). There were no difference of malondialdehyde (MDA) and the enzyme superoxide dismutase (SOD) between L-9 and A-16 before drought, while A-16 presented a prominent increase of MDA and significant decrease of SOD at the 4th day after drought treatment (Figure 1D,E).

**Figure 2:**
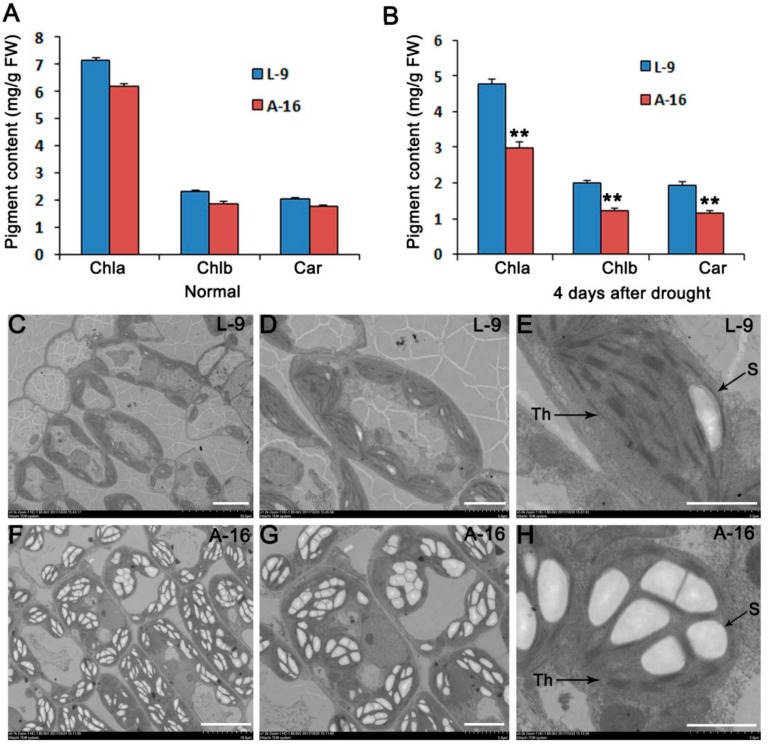
TEM observation of L-9 and A-16 leaves at seedling stage. (**A**) Chlorophyll content of L-9 and A-16 before drought. (**B**) Chlorophyll content of L-9 and A-16 during drought. Data is presented as the mean ± standard deviation (*n* = 9). ** *p* < 0.01; Student’s *t*-test. (**C**–**H**) Transmission electron microscopic photos of cells from L-9 and A-16. (**C**–**E**) Mesophyll cells in L-9 plants showed normal, well-ordered chloroplasts. (**F**–**H**) Cells in A-16 plants displayed some abnormalities and accumulated starch grains. Th: thylakoid, S: starch granule. Bar in (**C**,**F**): 100 μm. Bar in (**D**,**G**): 50 μm. Bar in (**E**,**H**) : 20 μm.

Before drought, there was no significant difference in chlorophyll content between L-9 and A-16 (Figure 2A). However, the relative content of chlorophyll a decreased to ~34% in L-9 vs. ~52% in A-16, and the chlorophyll b decreased to ~14% and ~33% in L-9 and A-16 after drought treatment, respectively (Figure 2B). These above results indicated that L-9 showed more significant drought tolerance than A-16. In order to compare the ultrastructure of chloroplasts between L-9 and A-16, we used the transmission electron microscopy to observe the leaves at seedling stage. The leaf cells of L-9 contained normal chloroplasts, which showed well-organized lamellar structures with normally stacked grana and thylakoid membranes (Figure 2C–E). However, most cells of A-16 were heteroplastidic, with many more starch grains (Figure 2F–H). These observations implied that the sensitivity to drought stress of A-16 might be related to the abnormal development of chloroplasts in leaves at the early seedling stage.

**Figure 3:**
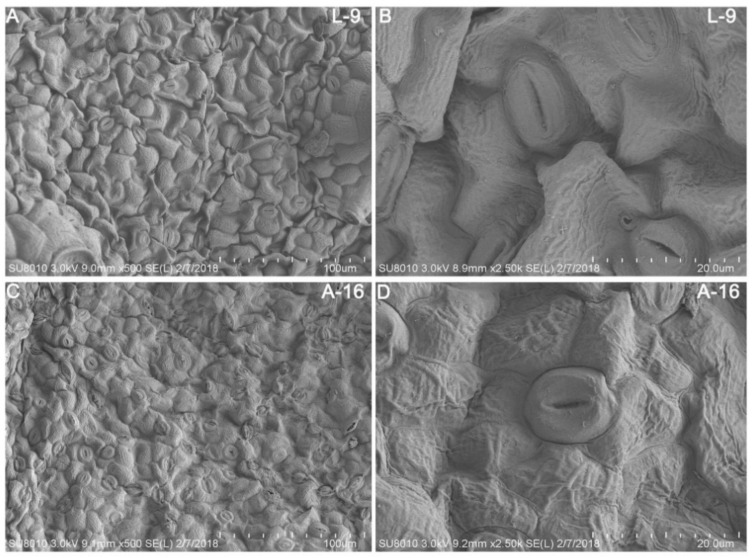
SEM observation of L-9 and A-16 leaves at seedling stage. (**A**,**B**) Scanning electron microscopy (SEM) images of leaves in L-9. (**C**,**D**) Scanning electron microscopy (SEM) images of leaves in A-16.

Additionally, we investigated whether stomatal numbers of A-16 was different from L-9 using scanning electron microscopy (SEM). The result showed that the number of stomas in L-9 (Figure 3A,B) was much less than A-16 (Figure 3C,D) in the same field size, indicating that L-9 lost water more easily when encountering drought stress.

### 2.2. Drought Stress Results in Extensive Transcriptomic Reprogramming

**Figure 4:**
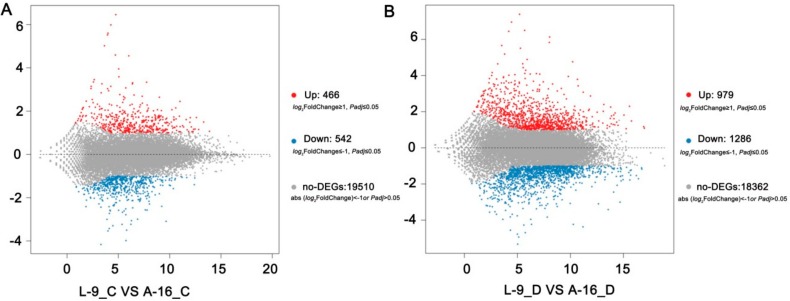
Comparison of different genes expression (DEGs) in leaves between L-9 and A-16 under normal conditions (**A**) and 4 days after drought (**B**). *x*- and *y*-axes represent log2 values of gene expression. Red, brown, and blue correspond to up-regulated, unaltered, and down-regulated gene expression, respectively. If a gene was expressed in just one sample, its expression value in another sample was replaced by the minimum value of all expressed genes in normal and drought samples. The screening threshold is given at the top of the figure.

**Figure 5:**
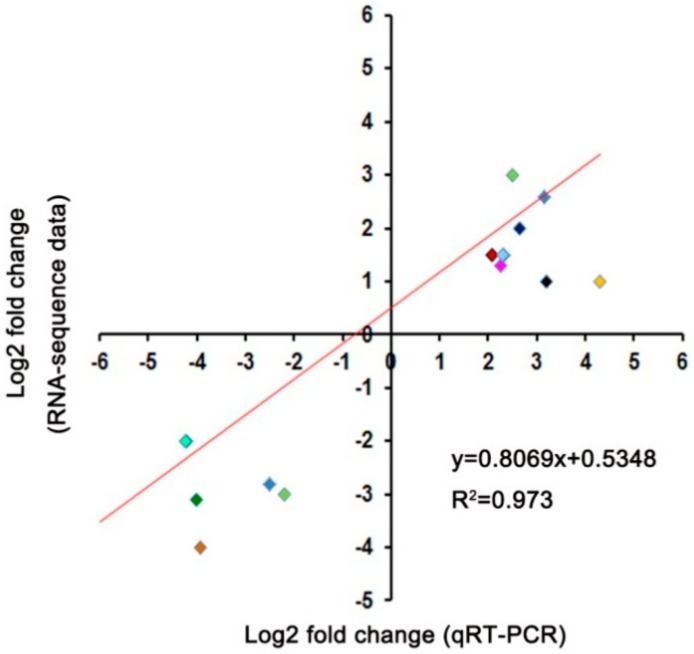
qRT-PCR validation of differentially expressed genes under drought stress. Correlation between the fold change analyzed by RNA-seq (*x*-axis) and data obtained using qRT-PCR. The different colors represent different genes expression.

In order to explore the transcriptional variations between L-9 and A-16 under normal and drought conditions, respectively, we carried out RNA-sequencing. A total of about 23 million clean reads were obtained per sample (Table 1) after removing the low-quality and adaptor-containing reads. At least 1.14 Gb clean data were acquired for each sample (Table 1). In total, the expression of 21,019 genes was detected. Approximately 96% of the clean reads were mapped to the reference cucumber genome [26], with more than 68% among them being uniquely mapped (Table 1). Finally, we identified 1008 (Table S1) and 2265 (Table S2) differentially expressed genes (DEGs) in the comparison of L-9 vs. A-16 under normal conditions and drought stress, respectively. Among them, under normal conditions, 466 genes were up-regulated and 542 down-regulated (gene expression in A-16 compared with L-9) (Figure 4A). Additionally, 979 up-regulated and 1286 down-regulated genes were identified during drought stress (Figure 4B). Next, in order to validate the RNA-seq results, we randomly selected 16 DEGs and conducted qRT-PCR analysis. The results showed that there was a strong positive correlation (two tailed, *R*^2^ = 0.973) between the RNA-seq and qRT-PCR result (Figure 5), which indicated the accuracy of the RNA-seq data.

### 2.3. Functional Classification of Drought-Responsive Genes

The gene ontology (GO) standardized classification system for gene function was used to analyze DEGs and understand the molecular events involved in drought response. Three categories, including “biological process,” “molecular function”, and “cellular components”, were classified under normal conditions (Figure S1A and Table S3) and drought stress (Figure S1B and Table S4), respectively. The number of the three category genes was prominently increased at 4 days after drought treatment, especially in the metabolic process, membrane, and catalytic activity, followed by subcategories such as cellular process, cell, and binding (Figure S1B).

**Figure 6:**
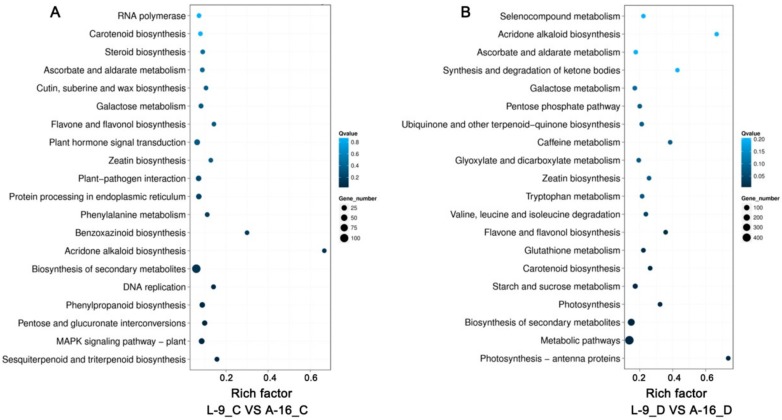
KEGG enrichment of annotated DEGs under three comparisons of normal conditions (**A**) and drought stress (**B**). The *y*-axis indicates the KEGG pathway and the *x*-axis indicates the enrichment factor. A high q-value is represented by light blue, and a low q-value is represented by dark blue.

Next, to examine DEG-associated pathways, they were searched in the KEGG pathway database. The top 20 enriched pathways are shown in Figure 6. The main pathways under normal conditions were “biosynthesis of secondary metabolites”, “plant hormone signal transduction”, and “MAPK signaling pathway” (Figure 6A and Table S5). When exposed to drought stress, genes related to “metabolic pathways” and “biosynthesis of secondary metabolites” were mostly enriched (Figure 6B and Table S6), indicating that these pathways and processes possibly participated in plant drought resistance. In addition, the category of “starch and sucrose metabolism” was only detected under stress conditions, suggesting these changed genes might contribute to the increased resistance of drought. Under water deficiency, we found that some genes were responsive to water deprivation (Table 2).

### 2.4. Expression of Genes Involved in Sucrose Biosynthesis and Response to Water Deprivation

**Figure 7:**
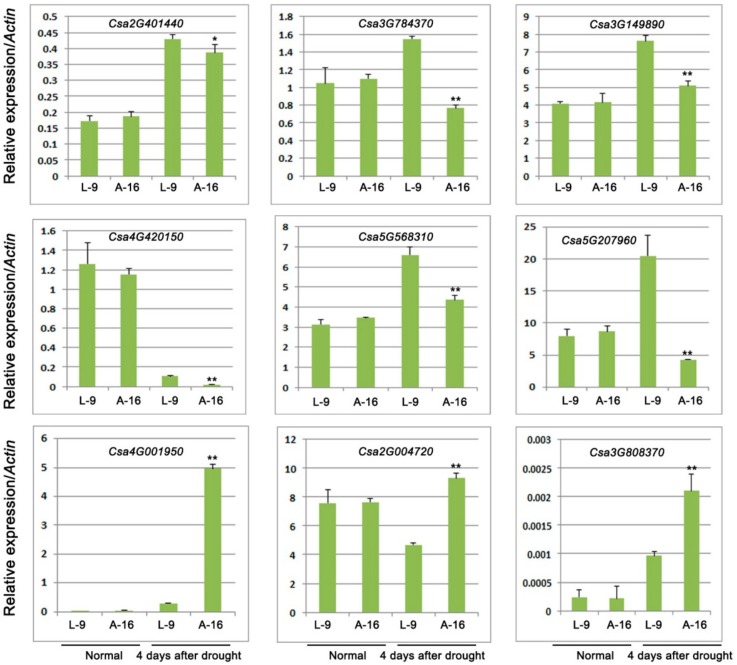
Relative expression of genes related to sucrose biosynthesis and response to water deprivation. Data is presented as the mean ± standard deviation (*n* = 9). * 0.01 ≤ *p* ≤ 0.05, ** *p* ≤ 0.01, Student’s *t* test.

Based on the results of GO and KEGG analysis, we chose several DEGs, which were involved in the starch and sucrose synthesis and response to drought stress. A total of 9 transcripts were selected, including 6 genes with sucrose or starch and 3 genes with response to water deprivation (Table 2 and Table S7). The qRT-PCR assay was employed to validate A-16 and L-9 of RNA-seq results under normal and drought stress, respectively. The results showed that no significant changes were detected between these two cultivars before treatment. However, when treated with drought stress for 4 days, six genes were significantly down regulated in A-16, especially genes involved in the sucrose metabolic process, sucrose biosynthetic process, and starch biosynthetic process. The expression of the remaining three genes including genes related to sucrose synthase activity and response to water deprivation, increased significantly in A-16 when compared with L-9 (Figure 7). These results of qRT-PCR were consistent with the RNA-sequencing data.

### 2.5. Analysis of Abscisic Acid (ABA)-Related Genes

**Figure 8:**
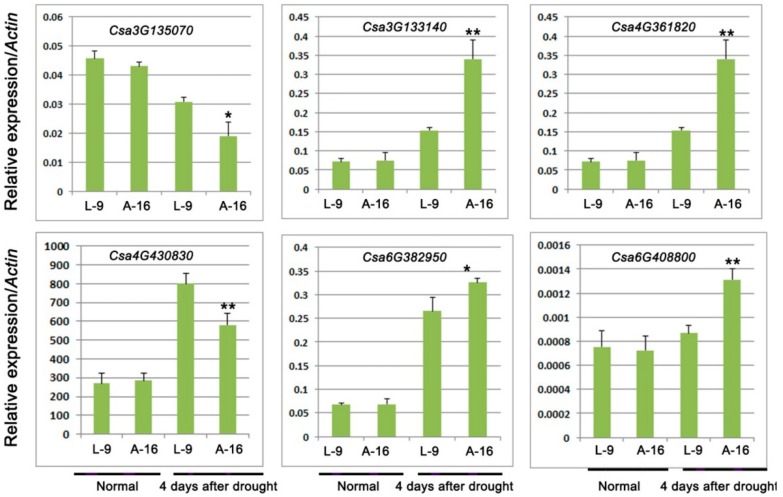
Relative expression of genes involved in ABA signaling pathway. Data is presented as the mean ± standard deviation (*n* = 9). * 0.01 ≤ *p* ≤ 0.05, ** *p* ≤ 0.01, Student’s *t* test.

Previous studies have reported that plant hormone, especially ABA, plays crucial roles in the regulation of the developmental process and signaling network involved in plant responses to drought stress [27]. Therefore, we selected the ABA-related genes among DEGs of drought stress from RNA-sequencing data (Table 3 and Table S7). In the present study, six genes related to the ABA signaling pathway were verified. The result showed that four genes were up-regulated and two down-regulated prominently (Figure 8), which was consistent with the RNA sequencing results.

## 3. Discussion

The analysis and availability of diverse genetic resources could offer important information for understanding the molecular basis of variability in their response to drought stress [16]. In the study, we characterized two cucumber genotypes for their significantly different response to drought (L-9 and A-16) stress. A-16 exerted drought sensibility under water deficiency with increased MDA content and decreased SOD enzyme activity and chlorophyll content. Through the analysis of the transcript level by RNA-seq, we found that the number of DEGs increased significantly at the 4th day after drought treatment. Among them, several DEGs related to the sucrose synthesis and ABA signaling pathway were possibly involved in the drought response tolerance with prominent expression changes between the two cultivars.

### 3.1. A-16 Has Less Stomata in the Leaf Than L-9

Previous studies have reported that the regulation of stomatal opening and closure is crucial to the normal transpiration and plays an important role in the resistance of drought stress [28]. In rice, *am1* mutant showed drought resistance and highly percentage of completely closed stomata when compared with the wild type [29]. Drought-tolerant variety *dca1* has a lower number of stomata and more completely closed stomata than the control [30]. In our present study, we found that the number of stomata in L-9 was less than in A-16 in the same field size, indicating that L-9 could enhance its tolerance to drought stress by regulating the number of stomata.

### 3.2. Analysis of Sucrose and Starch Biosynthetic Process in Drought Stress 

Sugar metabolism and starch biosynthesis are involved in the plant tolerance under drought stress [31]. Soluble sugar content is identified as a good marker in selecting the durum with drought tolerance [32]. The accumulation of soluble sugars in plant different tissues is reinforced when faced with different environmental stresses [33]. Under water deficiency, the soluble sugar was significantly accumulated in *Arabidopsis* leaves, resulting in its resistance to drought [34]. In our study, we found that most of genes involved in the sucrose and starch biosynthetic process were significantly up-regulated in the drought tolerant cultivar L-9, indicating that more sucrose and starch content might attribute to its resistance on drought stress.

### 3.3. Analysis of ABA Signal under Drought Stress

ABA plays essential role in the plant drought resistance because it could not only regulate the stomatal closure but also influence genes expression involved in stress-response and metabolic changes [35,36]. NAC transcriptional factors, which respond to ABA, could enhance plant tolerance under water deficiency [37,38,39]. In rice, both *OsNAC45* and *OsNAC52* were induced by ABA and their overexpressing transgenic plants showed enhanced tolerance to drought and salt treatments [38,40]. Here, we found that the expression of two genes (*Csa4M361820* and *Csa6M382950*), encoding the NAC domain-containing protein, increased prominently in the drought tolerant cultivar L-9, which was consistent with previous studies showing that higher expression of NAC genes could promote plant drought tolerance. Calcium-dependent protein kinase (CDPK), an important group of Ser/Thr protein kinases presents in plants and some protozoans that decode Ca^2+^ signals, are involved in the ABA signal transduction [41,42] and function in the plant response to drought [43,44]. Overexpression of *ZmCK3* (a maize calcium-dependent protein kinase gene) could improve plant survival rates under drought conditions in transgenic *Arabidopsis* [44]. CPK10, interacting with HSP1 (heat shock protein 1), plays important roles in ABA and Ca^2+^ mediated regulation of stomatal movements, leading to different tolerance to water deficiency [43]. *VfCPK1* of *Vicia faba* and *AtCPK11* of *Arabidopsis* are specifically induced by drought and ABA, respectively [45,46]. In this study, the drought-sensitive cultivar A-16 showed significantly decreased expression of *CDPK* genes (*Csa3M135070* and *Csa4M430830*) when compared with L-9, implying that high expression of *CDPK* might contribute to the drought tolerance in L-9.

### 3.4. Analysis of Cuticular Waxes Biosynthesis under Drought Stress

In addition, we also found that the “Cutin, suberin, and wax biosynthesis” pathway appeared under normal condition. The aerial surfaces of vascular plants are covered with a cuticle layer, including two major types of lipids, cutin and waxes [47]. Cuticular waxes play important roles in ensuring that plants grow and survive under various different biotic and abiotic stresses, which could help plants prevent non-stomatal water loss, and protect them against UV radiation and bacterial and fungal pathogens [48,49,50]. In the present study, several DEGs were enriched in the cutin pathway involved in lipid mechanism and were significantly down-regulated in A-16 when compared with L-9, suggesting that the decreased expression of related genes in cutin, suberin, and wax biosynthesis might be responsible for A-16’s sensitivity to drought stress. 

Overall, we firstly carried out RNA-Seq to analyze the regulation mechanism under water deficiency in cucumber. Several crucial genes involved in sucrose biosynthesis and ABA signal transduction were changed during drought stress. Our study not only provided a foundation for the further understanding of the regulation molecular on drought tolerance, but also explored valuable genes involved in drought tolerant, which will contribute to the improvement of drought resistant varieties in cucumber.

## 4. Materials and Methods

### 4.1. Plant Materials and Drought Treatment

Two cucumber cultivars, namely L-9 (South China type cucumber variety) and A-16 (North China type cucumber variety), were used in the study. Seeds were germinated overnight on wet filter in a culture dish at 28 °C in a dark environment. After that, the seedlings were grown in a feeding block under 14/10 h with 28/18 °C in day/night, respectively, in a culture room (5500 lux). When plants were grown to the two true leaves stage, they were subjected to lack of water for 7 days. After that, seedlings recovered for 3 days to normal condition. L-9 and A-16 seedlings were 120 for three biological replicates, respectively. Ten normal leaves were sampled from 10 plants before drought treatment, while drought-treated leaves were randomly sampled at the 4th day after drought treatment. Each biological replicate had a total of 10 leaves from 10 plants randomly selected. The samples were immediately frozen in liquid nitrogen and consistently stored at −80 °C until further analysis. In addition, leaf samples of three randomly selected biological replicates were then collected from both L-9 and A-16 plants (twelve samples in total).

### 4.2. Quantitative Analysis of Chlorophyll Content

Chlorophyll content was measured based on the procedure [51]. In detail, 0.2 g freshly-sampled leaves were homogenized in 5 mL solution with acetone and 0.1 M NH_4_OH at a ratio of 9:1 and then centrifuged at 3000× *g* for 20 min. The obtained supernatants were then washed three times using hexane and finally the pigment content was measured by spectrophotometer at the absorption wavelengths of 663 and 645 nm (Beckman Coulter DU-800, Brea, CA, USA). According to the two formulas (Ca = 13.95 × D_665_ − 6.88 × D_649_ × 6 (mg/L); Cb = 24.96 × D_649_ − 7.32 × D_665_ × 5 (mg/L)), the concentrations of chlorophyll a and chlorophyll b were finally calculated, respectively.

### 4.3. Analysis of Malondialdehyde (MDA) Content by TBA Method

MDA content was measured according to the following procedures. Briefly, 0.5 g freshly-sampled leaves were dipped into 0.5% trichloroacetic acid (TCA) and ground into powder, then centrifuged at 3000× *g* for 20 min. A total of 2 mL supernatant was added to 2 mL 0.5% thibabituric acid (TBA) 0.5% TCA, after that, the mixture was boiled at 100 °C for 30 min. Then, absorption wavelengths of supernatants on 450 nm, 532 nm, 600 nm were recorded. According to the given formula (CMDA = 6.45 × (A_532_ − A_600_) − 0.56 × A_450_ (μmol/L)), the MDA content was finally calculated.

### 4.4. Scanning Electron Microscopy (SEM)

Leaves of L-9 and A-16 seedlings under normal conditions were air-dried. The leaf abaxial epidermis was visualized under a HITACHI SU8020 variable pressure scanning electron microscope (SEM) (Hitachi, Tokyo, Japan) and imaged with an H-7500 transmission electron microscope (Hitachi).

### 4.5. Transmission Electron Microscopy (TEM)

Leaves of L-9 and A-16 seedlings under normal conditions were fixed overnight in 2.5% glutaraldehyde in 0.1 M phosphate buffer (pH 7.4) at 4 °C, then post-fixed in 2% (*v*/*v*) OsO4 in phosphate buffer. A series of 80 nm sections was cut using a Reichert OM2 ultramicrotome (Reichert, Deprew, New York, NY, USA), stained in 2% uranylacetate and 10 mM lead citrate (pH 12), before observation in a HitachiH-7650 (Hitachi) transmission electron microscope.

### 4.6. BGISEQ-500 Library Construction

A total of twelve samples (three biological replicates each of L-9 and A-16 at normal and drought stress, respectively) were used for RNA extraction with TRIZOL reagent according to the manufacturer’s protocol (TaKaRa, Shiga, Japan). Each biological replicate had a total of 10 leaves from 10 plants, selected randomly. After extraction, RNA was then purified (using DNAse) and concentrated using an RNeasyMinElute clean up kit (Qiagen, Duesseldorf, Germany). Then, 2.5 μg RNA of each sample was prepared for constructing BGISEQ-500 library according to the protocol of previous study [52]. Library quality was tested using the Agilent Bioanalyzer (Life Technologies, Carlsbad, CA, USA) 2100 system and the genome reference was the cucumber 9930 genome (http://cucurbitgenomics.org/, Two years).

### 4.7. Screening and Significant Test for Differentially Expressed Genes (DEGs)

Gene expression level was calculated by quantifying the reads according to the RPKM (reads per kilobase per million reads) method [53]. Then the NOISeq was used to identify DEGs, which existed in the normal and drought stress transcriptome libraries according to the following criteria: fold change ≥2 and divergence probability ≥0.8. GO enrichment for these DEGs was performed using WEGO software [54]. To further obtain knowledge of DEG biological functions, pathway enrichment analysis was carried out according to the KEGG database [55], the major public pathway-related database.

### 4.8. Quantitative Real-Time PCR (qRT-PCR) Identification

Quantitative real-time PCR analysis was performed using the total RNA from seedling leaves of both the normal and drought stress treatment. Twenty μL cDNA was obtained using the QuantiTect Reverse Transcription Kit (Qiagen, Duesseldorf, Germany). Quantitative qRT-PCR (20 μL reaction volume) was carried out with 0.5 μL of cDNA, 0.2 μM of primer mix and SYBR Premix Ex Taq Kit (TaKaRa,Shiga, Japan). In an ABI PRISM 7900HT system (Life Technologies, Carlsbad, CA, USA), cucumber α-TUBULIN (*TUA*) gene was used as normal. qRT-PCR was carried out on an ABI 7500 Real-Time PCR System (Applied Biosystems, USA). In addition, all qRT-PCR primers were listed in the Table S8.

### 4.9. Statistical Analysis

The linux rhel6.7 x64 R-3.4.2 and MEGA6 were used to perform the heat-map and cluster analysis. Significant differences were detected by IBM SPSS Statistics 20 (by Student’s *t* test). Relative gene expressions were calculated using the 2^−∆∆*C*t^ method [56]. In addition, GraphPad Prism 5 was used for chart preparation.

## Figures and Tables

**Table 1:** Mapping results of RNA sequencing reads of the cucumber between L-9 and A-16 under normal condition (C) and 4 days after drought (D).

Sample	Total Clean Reads	Total Clean Bases (Gb)	Total Mapping Ratio %	Uniquely Mapping Ratio %
A-16_C1	23,001,330	1.15	0.9664	0.8976
A-16_C2	22,799,582	1.14	0.9662	0.8986
A-16_C3	23,275,316	1.16	0.966	0.9013
A-16_D1	23,202,361	1.16	0.963	0.8943
A-16_D2	23,239,914	1.16	0.9625	0.8981
A-16_D3	23,127,940	1.16	0.959	0.8924
L-9_C1	23,343,741	1.17	0.966	0.8971
L-9_C2	23,065,366	1.15	0.9617	0.8901
L-9_C3	23,055,733	1.15	0.9634	0.8923
L-9_D1	22,973,680	1.15	0.9639	0.8927
L-9_D2	23,183,117	1.16	0.963	0.897
L-9_D3	23,037,373	1.15	0.956	0.8932

**Table 2:** Genes related to sucrose biosynthesis and response to water deprivation.

Gene ID	L-9 Expression	A-16 Expression	Regulation	*p*-Value	Annotation
*Csa2G401440*	2237.5	1091.6	Down	0.00434039	Sucrose-phosphate synthase
*Csa3G784370*	3412.9	1102.8	Down	1.20 × 10^−6^	Sucrose phosphatase
*Csa3G149890*	10,202.0	3362.5	Down	3.87 × 10^−12^	Glucose-1-phosphate adenylyltransferase
*Csa4G001950*	2345.5	7726.7	Up	6.84 × 10^−6^	Sucrose synthase
*Csa4G420150*	492.7	239.1	Down	1.17 × 10^−5^	4-α-Glucanotransferase
*Csa5G568310*	4872.5	2423.0	Down	3.78 × 10^−6^	Phosphoglucomutase
*Csa2G004720*	1255.1	2945.9	Up	1.42 × 10^−6^	Multiprotein-bridging factor
*Csa5G207960*	11,815.2	4338.2	Down	1.77 × 10^−9^	Omega-3 fatty acid desaturase
*Csa3G808370*	47.5	102.9	Up	0.00031088	Seed maturation protein LEA 4

**Table 3:** Genes involved in ABA signaling pathway.

Gene ID	L-9 Expression	A-16 Expression	Regulation	*p*-Value	Annotation
*Csa3G135070*	89.6	20.4	Down	3.05 × 10^−9^	Calcium-dependent protein kinase
*Csa3G133140*	463.1	1758.6	Up	1.72 × 10^−7^	3-Ketoacyl-CoA thiolase 1
*Csa4G361820*	1298.2	4273.3	Up	1.04 × 10^−17^	NAC domain-containing protein
*Csa4G430830*	276.8	50.0	Down	1.29 × 10^−18^	Calcium-dependent protein kinase-like protein
*Csa6G382950*	125.3	405.7	Up	4.08 × 10^−8^	NAC domain-containing protein
*Csa6G408800*	47.5	102.9	Up	0.0003109	Circadian clock coupling factor,

## Supplementary Materials

The following are available online at http://www.mdpi.com/1422-0067/19/7/2067/s1.

## Abbreviations

RNA seq	RNA sequencing
DEGs	Differently expressed genes
ABA	Abscisic acid
MDA	Malondialdehyde
SOD	Enzyme activity of superoxide dismutase
SEM	Scanning electron microscopy
TEM	Transmission electron microscopy
GO	Gene ontology
qRT-PCR	Quantitative Real-Time PCR
