# Supplementary material for: Transcriptome Analyses in Different Cucumber Cultivars Provide Novel Insights into Drought Stress Responses
Source: Int J Mol Sci. 2018 Jul 16;19(7):2067. doi: 10.3390/ijms19072067 (PMC6073345; doi:10.3390/ijms19072067)
Supplement: Supplementary file 1 [file ijms-19-02067-s001.zip › Supplemental materials/Figure S1.docx]

| 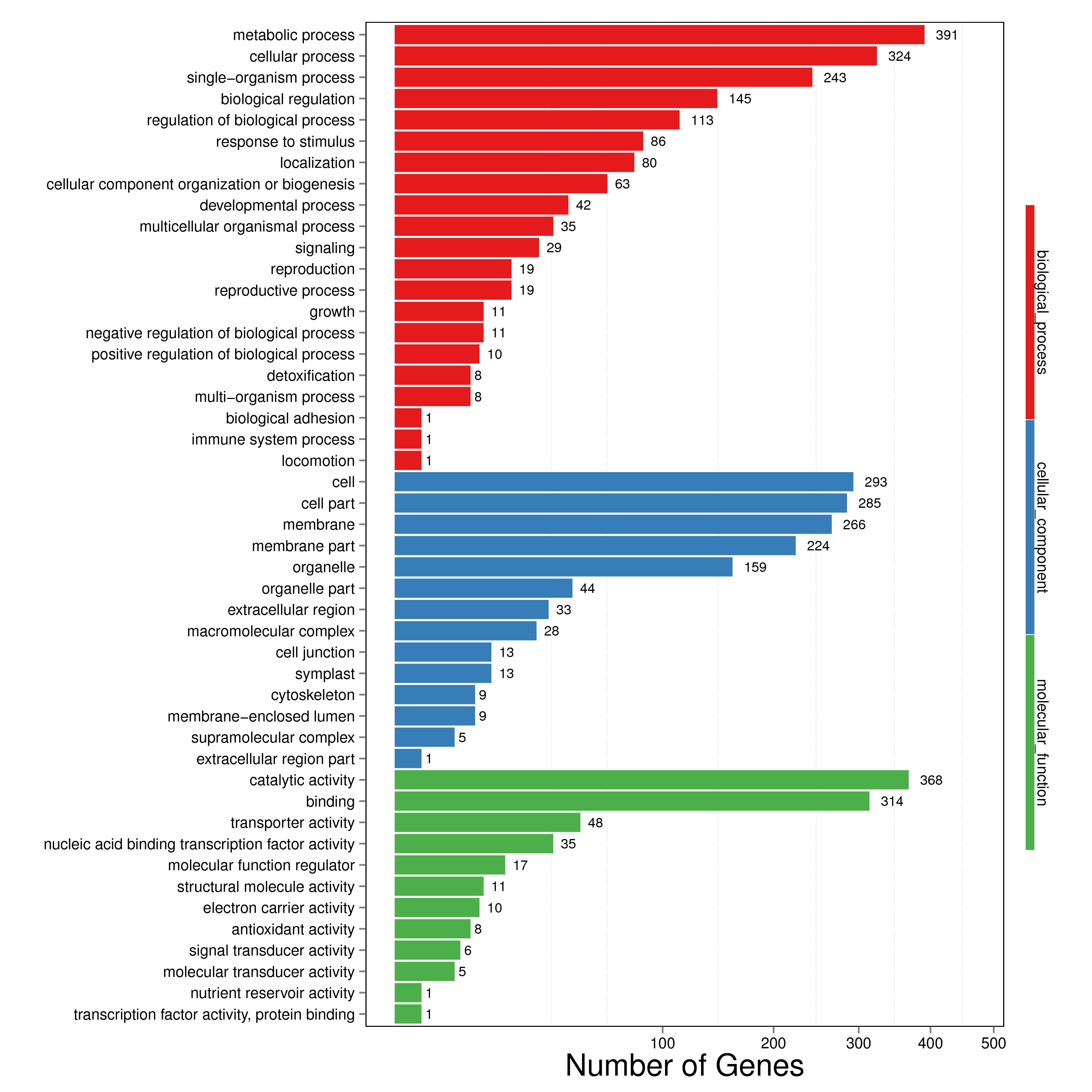 |
| --- |
| (A) |
| 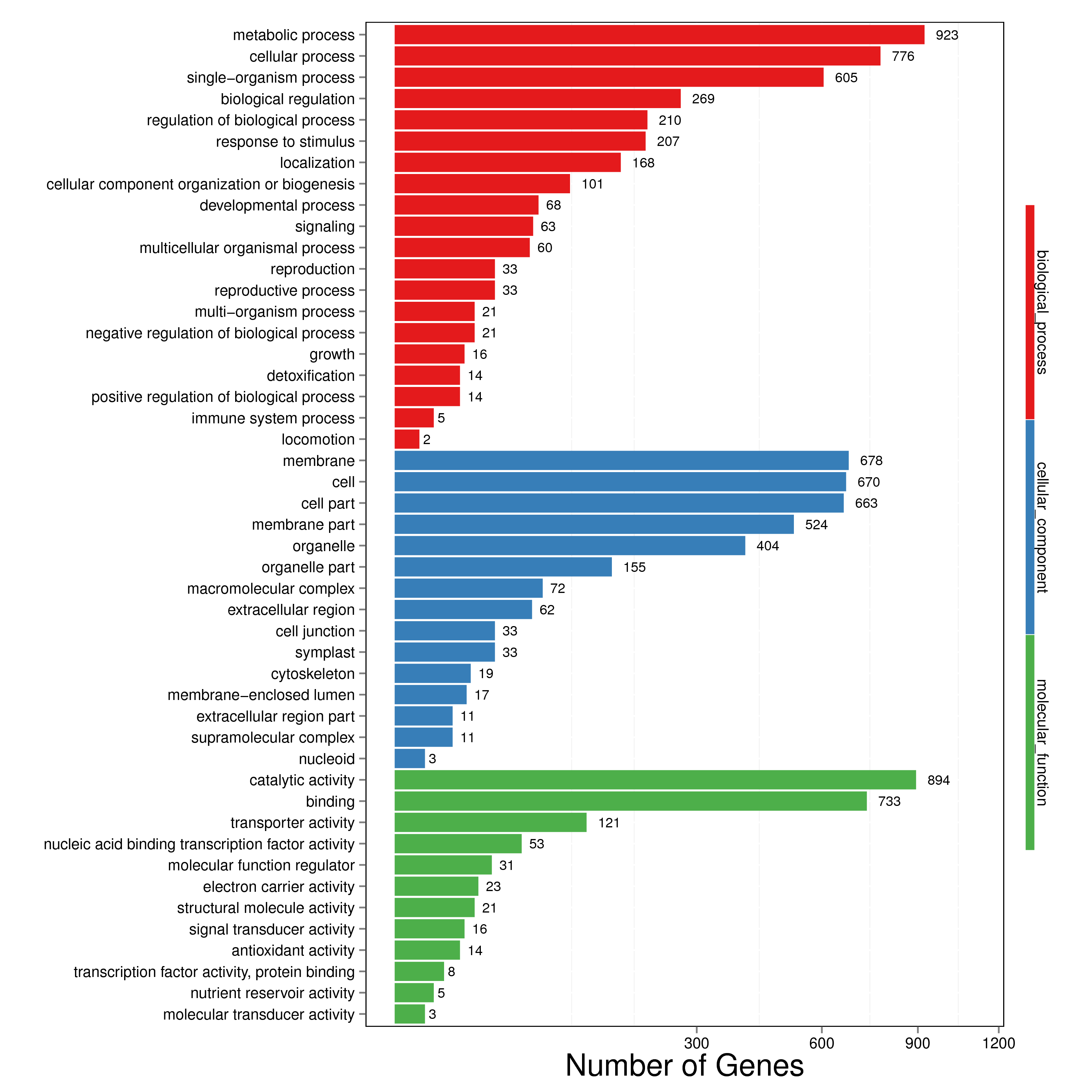 |
| (B) |

**Figure S1.** GO terms assigned to DEGs in leaves between L-9 and A-16 under normal condition (**A**) and 4 days after drought (**B**). The x-axis indicates the square root of the number of DEGs. The y-axis represents GO terms. All GO terms are grouped into three ontologies: red for biological process, blue for cellular component, and green for molecular function.
